# Practice patterns and factors influencing surgical trainees’ involvement in laparoscopic appendectomy in Northern Italy's largest educational network

**DOI:** 10.1007/s13304-025-02224-y

**Published:** 2025-05-16

**Authors:** Stefano Piero Bernardo Cioffi, Michele Altomare, Alessandra Borghi, Andrea Spota, Martino Bussa, Federico Ambrogi, Stefano Granieri, Francesco Virdis, The REsiDENT-1 research group, Stefania Cimbanassi

**Affiliations:** 1 Department of Surgery, University of Rome Sapienza, Rome, Italy; 2 General Surgery – Trauma Team, Niguarda Hospital, Piazzale Dell’Ospedale Maggiore 3, 20162 Milan, Italy; 3 Department of Surgery, Fondazione IRCCS Istituto Nazionale Dei Tumori, Milan, Italy; 4 Bicocca Bioinformatics, Biostatistics and Bioimaging Centre, School of Medicine and Surgery, University of Milano-Bicocca, Milan, Italy; 5 Department of Clinical Sciences and Community Health, University of Milan - La Statale, Milan, Italy; 6 Department of Pathophysiology and Transplantation, University of Milan – La Statale, Milan, Italy

**Keywords:** Surgical education, Laparoscopic appendectomy, Surgical residency, Learning curve

## Abstract

Introduction Laparoscopic Appendectomy (LA) allows residents to get early exposure to minimally invasive techniques. Despite its importance, involving residents as primary surgeons is a complex process, influenced by more than just clinical factors. Mentorship, environment, and perceptions play crucial roles. This study aimed to explore rates of residents' involvement in LA, the factors influencing the decision-making, and how surgeons and residents perceive the process. Methods We analyzed data from the REsiDENT-1 trial, covering 653 LA performed between 2019 and 2023 in 24 hospitals affiliated with the University of Milan. Adults with intraoperative acute appendicitis were included. Univariable analysis and multivariable logistic regression explored factors impacting residents’ involvement and clinical outcomes. A survey captured the perspectives of surgeons and residents. Results Residents approached 35.9% of the procedures, with longer operative times 67.14 (± 28.1) vs 71.68 (± 24.44), *p* = 0.001. Trainees were more involved in academic hospitals and emergency surgery units and less complex cases with lower complication rates. Residents' involvement was hampered by patients’ complexity in the multivariable analysis. Surgeons prioritized non-technical factors such as punctuality and reliability, whereas residents highlighted clinical complexity as a key consideration for the decision-making of the operator. Both groups agreed that structured feedback systems could improve the educational and training experiences. Conclusion LA remains a crucial procedure for surgical training, balancing hands-on learning with patient safety. Structured mentorship in academic and emergency settings could allow safe resident involvement. Future improvements should focus on clear feedback processes, better access to simulations, and standardized competency-based training to prepare residents for independent practice.

**Supplementary Information:**

The online version contains supplementary material available at 10.1007/s13304-025-02224-y.

## Introduction

Laparoscopic Appendectomy (LA) is often one of the first surgeries that trainees perform, giving them an early introduction to minimally invasive procedures [1]. Recent studies highlight the benefits of increasing residents’ responsibility, which helps them safely tackle more complex surgeries and enhances their learning experience [2]. When residents perform LA under appropriate conditions and with adequate supervision, patient outcomes remain unaffected [3], despite longer operative times [4].

However, understanding the involvement of residents in LA extends beyond clinical factors. A recent study from Norway highlighted that internal factors like a trainee’s basic skills, stress levels, and confidence significantly influenced their performance. External factors, such as the quality of mentorship and simulation tools, along with environmental pressures, also played a crucial role. The study concluded that trainees who were part of structured mentorship programs tended to perform better [5].

The European scenario on education in acute care surgery training is heterogeneous, due to differences inherent to each country. A common challenge is the lack of structured mentorship and standardized educational initiatives [6].

In Italy, surgical residency programs are heavily affected by young physician disaffections, due to environmental, quality of life, and educational constraints, mainly coming from the absence of a standardized approach to surgical education [7]. Despite this, scientific societies continue to invest considerable effort in implementing different educational programs to maximize the educational experience for aspiring surgeons covering all the subspecialties in the field of general surgery, including a new program focusing on acute care surgery [8].

What is still lacking is a comprehensive report exploring the trend of surgical trainees’ involvement in LA in the Italian scenario, understanding the dynamics beyond their involvement, and looking for educational gaps to address.

The study hypothesis was that residents’ involvement in LA is still scarce and that the decision-making process leading to residents’ involvement in LA is primarily influenced by non-technical and environmental factors rather than clinical ones, and that there are discrepancies in perceptions between surgeons and trainees on this process.

This study aimed to assess the rate of appendectomies approached by general surgery trainees and to shed light on the decision-making process behind the choice of the operator, assessing differences in perceptions, patients’ characteristics, and outcomes between surgeons and general surgery residents.

## Methods

### Primary endpoint

Evaluate the rate of LA approached by general surgery trainees in the clinical network of the residency program of the University of Milan.

### Secondary endpoints

- Identify factors associated with and predictors of trainees’ involvement in LA.
- Evaluate the perceptions of surgeons and trainees on the decision-making process leading to the selection of LA potentially approachable by a resident.

### Study design and setting

Data were reported according to the STROBE statement for observational studies [9].

Data were extracted from a four-year data lock of the REsiDENT-1 trial registry to perform a spin-off analysis. This is a multi-center project, started in October 2019, and approved by the ethics committee of the ASST GOM Niguarda Coordinating Center. Local registration number n° 486–22,072,021, ClinicalTrials.gov: NCT05075252. All the centers re-evaluated the protocol before the inclusion.

The REsiDENT-1 project aims to standardize the reporting of Acute Appendicitis (AA) severity and the grade of peritoneal contamination and explore the relationships between peritoneal irrigation and postoperative intra-abdominal abscesses [10].

Residents belong to the General Surgery Residency program of the University of Milan and do a clinical rotation every 6 months to 1 year within the clinical network of the University accounting for more than 50 surgical units. The online guidance regarding the compilation of the database was performed through group webinars led by seniors. A regular checkpoint and data cleaning are performed every 6 months by the steering committee. The list of residents and centers involved is available as supplementary material, S1.

### Patient enrolment

Inclusion criteria: age > 18 years, laparoscopic appendectomy (LA), and a histological diagnosis of AA. Exclusion criteria: conversion to open surgery and other primary causes of intra-abdominal infection clinically mimicking acute appendicitis (i.e. right colonic diverticulitis, gynaecological diseases).

Variables of interest in the registry included clinical, intraoperative, and postoperative data [10].

Acute appendicitis severity was reported following our published intraoperative classification [10]. Its clinical efficacy in identifying complex and simple diseases, using histology as the gold standard, is good to moderate and has been discussed in a previously published study [11]. The classification is reported below.

### Appendix aspect

- Erythematous and oedematous appendix
- Appendiceal phlegmon
- Gangrenous appendix
- Perforated appendix

### Contamination

- Single abscess
- Multiple abscesses
- Localized purulent peritonitis
- Diffuse purulent peritonitis
- Localized faecal peritonitis
- Diffuse faecal peritonitis

The presence of a complicated form of appendicitis was defined if at least one of the following was present: perforation or peritoneal contamination in any form.

The technical difficulty of LA was defined following a 5-point Likert-type scale [12], considering the progressive operative autonomy of the operator from a procedure performed independently (1), via procedures requiring passive (2) and active (3) assistance from the assistant, to complex, challenging procedures needing an external, non-scrubbed (4) or scrubbed (5), help to finish the LA.

The perceptions of residents and surgeons on the decision-making were explored via a web-based survey. This electronic survey study was performed in October 2023 at the end of the data lock on the REsiDENT-1 registry. The population of interest consisted of trainees who joined the study and performed data collection and surgeons working in the same centers as the residents involved in the study. Participation was anonymous, confidential, voluntary, and free from financial incentives. Thus, we did not request Institutional Review Board (IRB) study approval.

### Survey design

A 15-question electronic survey instrument was developed by the steering committee of the REsiDENT-1 study along with representatives of the scientific committee of the General Surgery residency program of the University of Milan. Because no suitable validated survey instruments exist, we created a survey instrument to qualitatively explore the perceptions on the decision-making process leading to the choice of the operator of an LA. The survey was divided into four parts exploring: respondent demographics, the role of clinical factors, the role of environmental factors, and the role of non-technical factors. The complete survey is reported in the Supplementary materials, S2. The survey was conducted following the CHERRIES (Checklist for Reporting Results of Internet E-Surveys) method [13]. A practical guide published in JAMA Surgery on survey research was also followed [14]. The survey was administered through RedCAP [15] and was delivered via email to the 69 residents involved in data collection and to 250 surgeons working in the 24 units involved in the study.

### Survey data management

To prevent multiple data entries from the same respondent the IP address was used to automatically identify potential duplicates.

### Study data collection and storage

Data was anonymized and stored securely in an online secure database. [15]

## Statistical analysis

### Primary endpoint analysis

The primary endpoint analysis was performed with descriptive statistics.

Numeric variables are expressed as mean (± SD) and discrete outcomes as absolute and relative (%) frequencies.

### Secondary endpoints analysis

The first of the secondary endpoints was explored with two statistical approaches to explore associated factors with surgeries performed by a resident or a surgeon.

- Continuous outcomes were compared with unpaired Student t-test, Welch t-test, or Mann–Whitney U test according to data distribution. Discrete outcomes were compared with chi-squared or Fisher’s exact test accordingly. We created 2 groups according to the operator approaching the LA, Resident, or Surgeon. Group comparability was assessed by comparing baseline demographic and clinical data. Normality and heteroskedasticity of continuous data were assessed with Shapiro–Wilk and Levene’s test, respectively.. The alpha risk was set to 5% and two-tailed tests were used.
- A multivariable logistic regression model was developed with the operator (surgeon or resident) as the dependent variable, using the resident as the reference category. Pre-operative variables included in the model were selected based on univariate analysis results and their clinical relevance. Data were checked for multicollinearity using the Bels- ley–Kuh–Welsch technique. Heteroskedasticity and normality of residuals were assessed using the White test and Shapiro–Wilk test, respectively. The confidence interval (CI) was set at 95%. The alpha risk was set at 5% and two-tailed tests were used.

To study the second and last secondary endpoint a univariable analysis was performed comparing the responses to the survey administered to surgeons and residents involved in the surgical units who joined the study. Group comparability was assessed by comparing baseline demographic data and follow-up duration between groups. Normality and heteroskedasticity of continuous data were assessed with the Shapiro–Wilk and Levene’s test, respectively. Continuous outcomes were compared with unpaired Student t-test, Welch t-test, or Mann–Whitney U test according to data distribution. Discrete outcomes were compared with chi-squared or Fisher’s exact test accordingly. The alpha risk was set to 5% and two-tailed tests were used.

Statistical analyses were performed using EasyMedStat (version 3.20.4; www.easymedstat.com) and R software (R Core Team (2021). R: A language and environment for statistical computing. R Foundation for Statistical Computing, Vienna, Austria. URL https://www.R-project.org/).

## Results

### Primary endpoint analysis

Surgical trainees approached LA in 35.9% of the procedures, 234/653. The median number of LA performed by residents at the time of the study was 11 (IQR 6–20). Among the residents who approached the LA as a first operator, 29.5% (69/234) had already performed more than 20 LA. The results of the demographic analysis are available in Table 1.

**Table 1:** Demographics of the general population. EGS, Emergency General Surgery. BMI, Body Mass Index. ASA, American Society of Anesthesiologists

Variable	Mean (SD)	95% CI	Min	Q1	Median
	Count (%)		Max	Q3	
Hospital type (1 University 2 Non university)					
No	417 (63.9)	0.602			
		0.675			
Yes	236 (36.1)	0.325			
		0.398			
Dedicated EGS service					
No	458 (70.1)	0.666			
		0.736			
Yes	195 (29.9)	0.264			
		0.334			
Age	37.74 (16.37)	36.49	**18**	23	35
		39	**91**	50	
BMI	24.31 (3.89)	24.01	**15**	22	24
		24.61	**42**	26.4	
ASA					
1	378 (58.0)	0.542			
		0.618			
2	238 (36.5)	0.328			
		0.402			
3	34 (5.2)	0.035			
		0.069			
4	2 (0.3)	0			
		0.007			
Charlson Comorbidity Index	0.528 (1.15)	0.439	**0**	0	0
		0.616	**11**	1	
PIRO score	0.248 (0.741)	0.191	**0**	0	0
		0.305	**9**	0	
Alvarado Score	6.49 (1.68)	6.36	**1**	5	7
		6.61	**10**	8	
AIR score	5.89 (2.0)	5.73	**1**	4	6
		6.04	**12**	7	
Point Of Care Ultrasound					
No	621 (95.1)	0.934			
		0.968			
Yes	32 (4.9)	0.032			
		0.066			
US performed by a radiologist					
Yes	352 (53.9)	0.501			
		0.577			
No	301 (46.1)	0.423			
		0.499			
CT Scan					
No	373 (57.1)	0.533			
		0.609			
Yes	280 (42.9)	0.391			
		0.467			
White Blood Cells (^3)	13.86 (4.51)	13.51	**1.58**	11	13.64
		14.21	**58.3**	16.3	
Neutrophils (%)	78.89 (11.12)	77.88	**15.37**	75	80.3
		79.89	**98**	86	
C Reative Protein (mg/dl)	6.83 (7.74)	6.24	**0**	1.12	4.32
		7.43	**46.2**	9.52	
Time to surgery (hour)	16.74 (18.28)	15.34	**0.95**	8	13
		18.15	**360**	22	
Operative time (minutes)	68.77 (26.91)	66.7	**15**	50	60
		70.84	**230**	82	
Operator 1 Resident 2 Surgeon					
2	418 (64.1)	0.604			
		0.678			
1	234 (35.9)	0.322			
		0.396			
Number of lap appy performed by the resident	14.89 (11.8)	13.26	**0**	6	11
		16.52	**60**	20.25	
Operator 1 Resident < 20 LA 2 Resident > 20 LA 3 Surgeon					
3	418 (64.0)	0.603			
		0.677			
1	135 (20.7)	0.176			
		0.238			
2	69 (10.6)	0.082			
		0.129			
0	31 (4.7)	0.031			
		0.064			
Difficulty grade					
1	141 (21.6)	0.185			
		0.248			
2	236 (36.2)	0.325			
		0.399			
3	165 (25.3)	0.22			
		0.286			
4	87 (13.3)	0.107			
		0.16			
5	18 (2.8)	0.015			
		0.04			
Aspect of the appendix 1 erythematous 2 phlegmpn 3 gangrenous 4 perforated					
1	109 (16.7)	0.139			
		0.196			
2	337 (51.7)	0.479			
		0.555			
3	148 (22.7)	0.195			
		0.259			
4	58 (8.9)	0.067			
		0.111			
Complicated Appendicitis (perforation or peritoneal contamination)					
Yes	372 (57.1)	0.533			
		0.609			
No	280 (42.9)	0.391			
		0.467			
Peritoneal Irrigation					
Yes	485 (74.4)	0.71			
		0.777			
No	167 (25.6)	0.223			
		0.29			
Drainage					
Yes	348 (53.4)	0.495			
		0.572			
No	304 (46.6)	0.428			
		0.505			
Length of Stay (days)	4.19 (2.06)	4.03	**0**	3	4
		4.35	**21**	5	
Intensive Care Unit					
No	634 (97.4)	0.962			
		0.986			
Yes	17 (2.6)	0.014			
		0.038			
Duration of antimicrobial therapy	6.29 (2.76)	6.04	**0**	4	6
		6.53	**21**	8	
Clavien Dindo classification of surgical complications	0.499 (0.877)	0.432	**0**	0	0
		0.567	**7**	1	
Death					
No	651 (99.7)	0.993			
		1.001			
Yes	2 (0.3)	0			
		0.007			
Health-care related expenditures	3330.18 (945.22)	3257.55	**1044**	2560	3514
		3402.81	**5735**	3514	
New hospitalization at 30 days					
No	498 (97.3)	0.959			
		0.987			
Yes	14 (2.7)	0.013			
		0.041			
New hospitalization at 60 days					
No	511 (99.8)	0.994			
		1.002			
Yes	1 (0.2)	0			
		0.006			
New hospitalization at 90 days					
No	512 (100.0)	1			
		1			
Yes	0 (0.0)	0			
		0			
Unplanned readmission					
No	497 (97.1)	0.956			
		0.985			
Yes	15 (2.9)	0.015			
		0.044			
Surgical Site Infections					
No	607 (93.0)	0.91			
		0.949			
Yes	46 (7.0)	0.051			
		0.09			
Organ Space Surgical Site Infections					
No	617 (94.5)	0.927			
		0.962			
Yes	36 (5.5)	0.038			
		0.073			

**Fig. 1:**
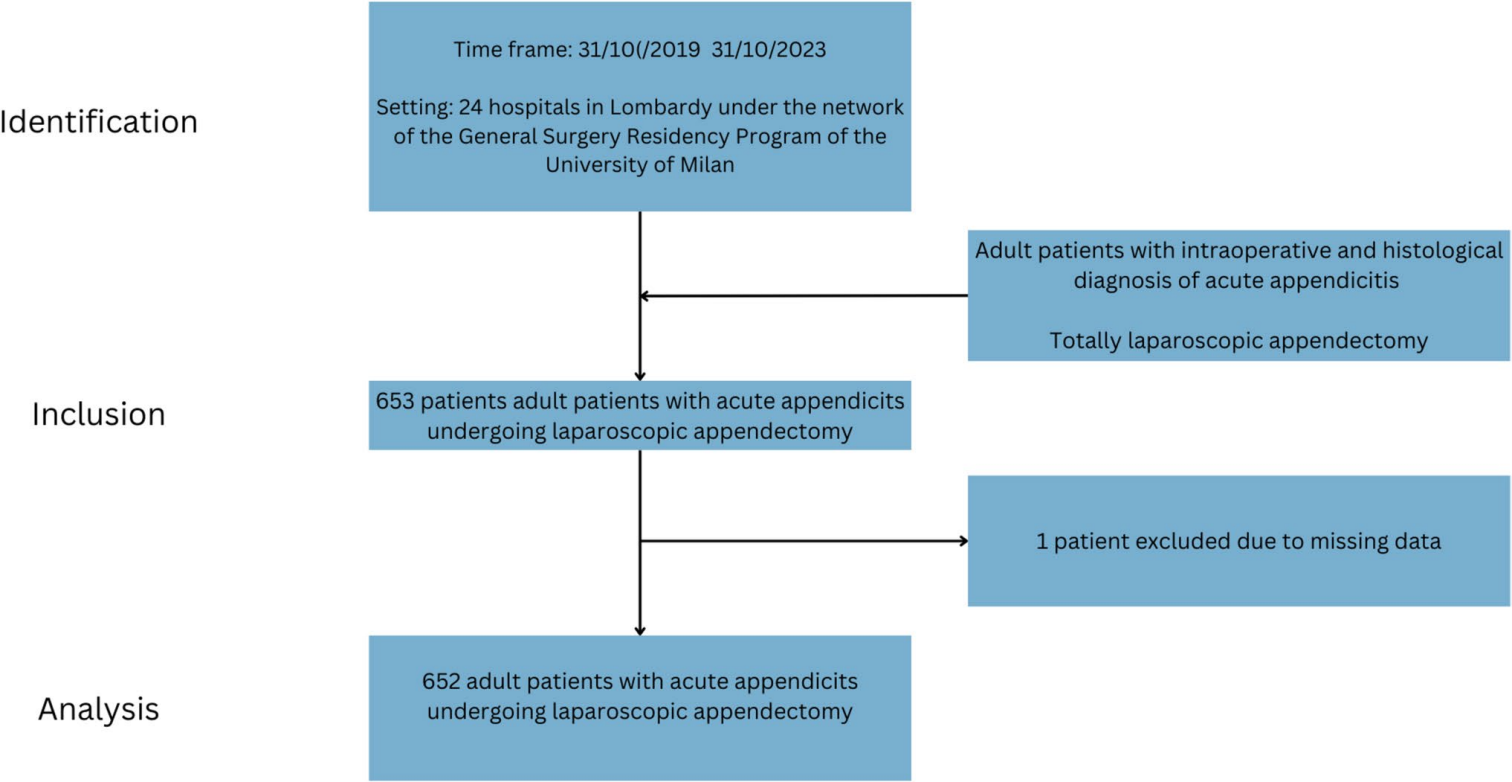
Flowchart of patients’ enrolment according to the STROBE checklist of cohort studies

The flowchart of patients enrolment is available in Fig. 1.

### Secondary endpoint analysis

The univariable analysis compared patients undergoing LA approached by residents and surgeons. Residents were more frequently involved as first surgeons in LA in university hospitals, 57.3% vs 24.2%, *p* < 0.001, and dedicated emergency general surgery units, 51.3% vs 17.9%, *p* < 0.001. Residents were primary surgeons in patients with lower ASA scores, *p* = 0.02, with less comorbidities, Charlson Comorbidity Index 0.38 vs 0.69, *p* = 0.002. In addition, when no diagnostic was performed surgeons were more prone to approach the LA, *p* = 0.05. The rate of gangrenous and perforated appendicitis was also lower for residents, *p* = 0.004 as well as the rates of peritoneal contamination, *p* < 0.001. The reported difficulty grade was overall higher in LA approached by surgeons, 18.7% vs 12.6% for grades 4 and 5 in which external help was necessary to complete the procedure. The Clavien Dindo classification of surgical complications was significantly higher for all grades above 1, *p* = 0.002, but no difference was identified for grades 3–4-5.

Residents had longer operative times, 67.14 (± 28.1) vs 71.68 (± 24.44), *p* = 0.001, less use of peritoneal irrigation, 64.5% versus 79.9%, *p* = 0.011, intra-abdominal drainages, 38.5% versus 61.7%, *p* < 0.001, and shorter duration of the postoperative antimicrobial therapy, when administered, 5.5 (± 2.58) versus 6.7 (± 2.7), *p* = 0.002, and of the total length of the hospital stay 3.7 (± 1.69) versus 4.1 (± 1.74), *p* = 0.002. The full results of the analysis are available in Table 2.

**Table 2:** Results of the univariable analysis. BMI, Body Mass Index. ASA, American Society of Anaesthesiologists score. CCI, Charlson Comorbidity Index. SSI, Surgical Site Infection. OS SSI, Organ Space Surgical Site Infection

Variable	Surgeon N = 418	Resident N = 234	p-Value
Hospital type University Non University	101 (24.16%) 317 (75.84%) N = 418	134 (57.26%) 100 (42.74%) N = 234	< 0.001
Dedicated EGS service Yes No	75 (17.94%) 343 (82.06%) N = 418	120 (51.28%) 114 (48.72%) N = 234	< 0.001
Year of residency 1 2 3 4	3 (8.11%) 2 (5.41%) 16 (43.24%) 16 (43.24%) N = 37	3 (5.56%) 4 (7.41%) 31 (57.41%) 16 (29.63%) N = 54	0.469
Age	38.53 (± 16.77) Range: (18.0; 87.0) N = 418	36.36 (± 15.58) Range: (18.0; 91.0) N = 234	0.162
BMI	24.38 (± 4.05) Range: (15.0; 42.0) N = 410	24.17 (± 3.58) Range: (15.9; 38.5) N = 228	0.807
ASA 1 2 3 4	212 (50.84%) 177 (42.45%) 26 (6.24%) 2 (0.48%) N = 417	165 (70.51%) 61 (26.07%) 8 (3.42%) 0 (0.0%) N = 234	< 0.001
Charlson Comorbidity Index	0.609 (± 1.23) Range: (0.0; 11.0) N = 417	0.385 (± 0.979) Range: (0.0; 6.0) N = 234	0.002
PIRO score	0.314 (± 0.855) Range: (0.0; 9.0) N = 417	0.132 (± 0.458)Range: (0.0; 3.0)N = 234	< 0.001
Alvarado Score	6.49 (± 1.67) Range: (1.0; 10.0) N = 417	6.48 (± 1.7) Range: (2.0; 10.0) N = 234	0.966
AIR score	5.93 (± 1.97) Range: (1.0; 12.0) N = 392	5.81 (± 2.04) Range: (1.0; 11.0) N = 227	0.419
Point Of Care Ultrasound Yes No	25 (5.98%) 393 (94.02%) N = 418	7 (2.99%) 227 (97.01%) N = 234	0.132
US performed by a radiologist Yes No	206 (49.28%) 212 (50.72%) N = 418	146 (62.39%) 88 (37.61%) N = 234	0.002
CT ScanYesNo	186 (44.5%) 232 (55.5%) N = 418	93 (39.74%) 141 (60.26%) N = 234	0.274
No imaging performed Yes No	36 (8.61%) 382 (91.39%) N = 418	10 (4.27%) 224 (95.73%) N = 234	0.055
White Blood Cells (^3)	14.06 (± 4.28) Range: (3.0; 27.8) N = 418	13.5 (± 4.87) Range: (1.58; 58.3) N = 234	0.132
Neutrophils (%)	79.38 (± 11.11) Range: (15.37; 98.0) N = 305	78.0 (± 11.11) Range: (20.15; 95.1) N = 167	0.183
C Reactive Protein (mg/dl)	7.55 (± 8.49) Range: (0.0; 46.2) N = 418	5.56 (± 6.0)Range: (0.0; 35.0)N = 234	0.045
Platelets (^3)	251.56 (± 76.03) Range: (16.0; 676.0) N = 418	246.06 (± 58.37) Range: (23.0; 407.0) N = 234	0.886
INR ratio	1.16 (± 0.291) Range: (0.8; 5.6) N = 418	1.11 (± 0.155) Range: (0.3; 2.2) N = 234	0.015
Time to surgery (hour)	16.37 (± 13.07) Range: (0.95; 120.0) N = 418	17.41 (± 25.06) Range: (2.0; 360.0) N = 233	0.881
Operative time (minutes)	67.14 (± 28.1) Range: (15.0; 230.0) N = 418	71.68 (± 24.44) Range: (20.0; 180.0) N = 233	0.001
Difficulty grade 0 1 2 3 4 5	3 (0.72%) 91 (21.77%) 151 (36.12%) 95 (22.73%) 69 (16.51%) 9 (2.15%) N = 418	2 (0.85%) 50 (21.37%) 85 (36.32%) 70 (29.91%) 18 (7.69%) 9 (3.85%) N = 234	0.02
Aspect of the appendix Erythematous Phlegmon Gangrenous Perforated	61 (14.59%) 205 (49.04%) 109 (26.08%) 43 (10.29%) N = 418	48 (20.51%) 132 (56.41%) 39 (16.67%) 15 (6.41%) N = 234	0.004
Peritoneal contamination Fecal Peritonitis No Contamination Purulent peritonitis Single/Multiple Abscess	21 (5.02%) 170 (40.67%) 144 (34.45%) 83 (19.86%) N = 418	32 (13.68%) 111 (47.44%) 52 (22.22%) 39 (16.67%) N = 234	< 0.001
Peritoneal Irrigation Yes No	334 (79.9%) 84 (20.1%) N = 418	151 (64.53%) 83 (35.47%) N = 234	< 0.001
Volume of irrigation	1052.19 (± 1469.09) Range: (30.0; 10,000.0) N = 310	692.68 (± 795.6) Range: (0.0; 5000.0) N = 142	0.01
Drainage Yes No	258 (61.72%) 160 (38.28%) N = 418	90 (38.46%) 144 (61.54%) N = 234	< 0.001
Length of Stay (days)	4.47 (± 2.2) Range: (1.0; 21.0) N = 415	3.7 (± 1.69) Range: (0.0; 13.0) N = 233	< 0.001
Intensive Care Unit Yes No	14 (3.35%) 404 (96.65%) N = 418	3 (1.29%) 230 (98.71%) N = 233	0.131
Postoperative antimicrobial therapy Yes No	329 (78.71%) 89 (21.29%) N = 418	163 (69.96%) 70 (30.04%) N = 233	0.017
Antimicrobial regimen	4.08 (± 3.4) Range: (1.0; 12.0) N = 333	3.03 (± 3.05) Range: (1.0; 12.0) N = 166	< 0.001
Duration of antimicrobial therapy	6.7 (± 2.76) Range: (0.0; 21.0) N = 317	5.5 (± 2.58) Range: (0.0; 14.0) N = 166	< 0.001
Clavien Dindo classification of surgical complications 0–1 > 1	351 (83.97%) 67 (16.03%) N = 418	217 (92.74%) 17 (7.26%) N = 234	0.002
Clavien Dindo classification of surgical complications 0-2 3–4-5	411 (98.33%) 7 (1.67%) N = 418	232 (99.15%) 2 (0.85%) N = 234	0.501
Death Yes No	2 (0.48%) 416 (99.52%) N = 418	0 (0.0%) 234 (100.0%) N = 234	0.539
Health-Care related expenditures	3419.62 (± 1002.19) Range: (1044.0; 5735.0) N = 418	3173.71 (± 813.06) Range: (1044.0; 5735.0) N = 234	0.002
Unplanned readmission at 90 days Yes No	8 (2.41%) 324 (97.59%) N = 332	7 (3.89%) 173 (96.11%) N = 180	0.412
Length of Stay for new hospitalization	1.6 (± 3.37) Range: (0.0; 11.0) N = 35	0.9 (± 3.02) Range: (0.0; 17.0) N = 50	0.395
SSI overall Yes No	30 (7.18%) 388 (92.82%) N = 418	16 (6.84%) 218 (93.16%) N = 234	0.998
OS SSI overall Yes No	23 (5.5%) 395 (94.5%) N = 418	13 (5.56%) 221 (94.44%) N = 234	> 0.999

In the multivariable analysis, the odds of the surgeon initiating the procedure increased by 120% when no imaging diagnostics were performed (OR = 2.22, 95% CI = 1.06–4.7), controlling for age, sex, CRP levels, and ASA risk class. Among patients classified as ASA 2, the odds of the surgeon initiating the procedure increased by 153% compared to patients classified as ASA 1 (OR = 2.53, 95% CI = 1.69–3.78). For patients with ASA classifications of 3–4, the odds of the surgeon initiating the procedure were nearly four times higher compared to ASA 1 patients (OR = 3.879, 95% CI = 1.55–9.699). The results of the model are summarized in Table 3.

**Table 3:** Results of the multivariable logistic regression model. The dependent variable is the operator (surgeon versus resident). ASA, American Society of Anaesthesiologists. CRP, C Reactive Protein

	Adjusted Odd Ratio	95% Confidence Interval	P value
Age	0.99	0.98–1.00	0.098
Female Sex (Reference Male)	1.43	1.03–2.00	0.035
No diagnostic imaging	2.22	1.06–4.65	0.034
CRP	1.04	1.01–1.06	0.005
ASA = 2 (Reference ASA = 1)	2.53	1.69–3.78	0.001
ASA = 3–4 (Reference ASA = 1)	3.88	1.55.9–70	0.004

The second secondary endpoint was explored by comparing surgeon and resident responses to the survey administered. The survey response rate was 26%, 83/319. Sixty-eight point-one percent, 47/69, among residents and 14.4%, 36/250 among surgeons. Trainees prioritized clinical factors, such as the presence of complicated disease, 25.5% versus 8.3%, *p* < 0.05, in the decision-making process.

Surgeons prioritized non-technical and environmental factors such as the year of residency. 63.1% vs 44.7%, *p* = 0.015, and punctuality and reliability, 47.2%vs.23.4%, *p* = 0.041.

Both groups agreed that a standardized perioperative feedback system would improve the process of residents’ involvement in LA. The full comparison is available in Table 4.

**Table 4:** Results of the survey showing group comparison exploring residents and surgeon perceptions on the decision-making beyond residents involvement in laparoscopic appendectomy

Variable	General surgery resident	Surgeon	p-Value
	N = 47	N = 36	
In which kind of hospital do you work? Community hospital Large referral centre Large referral university centre	13 (27.66%) 14 (29.79%) 20 (42.55%) N = 47	8 (22.22%) 8 (22.22%) 20 (55.56%) N = 36	0.498
Does you hospital have a dedicate emergency surgery service? Yes No	36 (76.6%) 11 (23.4%) N = 47	21 (58.33%) 15 (41.67%) N = 36	0.124
Do you believe that the year of residency is a determining factor to let a resident operate on a Laparoscopic Appendectomy?	44.7 (± 31.98) Range: (0.0; 100.0) N = 47	63.11 (± 30.55) Range: (5.0; 100.0) N = 36	0.015
How important do you think is the number of surgeries previously performed by a resident to be considered ready to perform a laparoscopic appendectomy? 1 2 3 4 5	7 (14.89%) 11 (23.4%) 11 (23.4%) 11 (23.4%) 7 (14.89%) N = 47	1 (2.78%) 6 (16.67%) 9 (25.0%) 11 (30.56%) 9 (25.0%) N = 36	0.282
Do you think that a Laparoscopic Appendectomy should be performed by a resident only if s/he has performed a certain number of laparoscopic procedures other than laparoscopic appendectomy? Yes No	4 (8.51%) 43 (91.49%) N = 47	7 (19.44%) 29 (80.56%) N = 36	0.196
How many laparoscopic procedures should have been performed by a resident to safely involve her/him into a Laparoscopic Appendectomy? 11 to 15 16 to 20 5 to 10 Less than 5 More than 20	0 (0.0%) 1 (25.0%) 1 (25.0%) 1 (25.0%) 1 (25.0%) N = 4	2 (28.57%) 2 (28.57%) 3 (42.86%) 0 (0.0%) 0 (0.0%) N = 7	0.509
How many laparoscopic appendectomies should a surgeon have performed to be ready to assist a resident during a laparoscopic appendectomy? 20 21–30 31-40 41-50 Less than 20 The number doesn’t matter more than 50	7 (14.89%) 6 (12.77%) 12 (25.53%) 8 (17.02%) 1 (2.13%) 4 (8.51%) 9 (19.15%) N = 47	7 (19.44%) 7 (19.44%) 10 (27.78%) 3 (8.33%) 1 (2.78%) 1 (2.78%) 7 (19.44%) N = 36	0.81
Do you believe that only > 40 years old surgeons should assist a resident during a laparoscopic appendectomy?	10.72 (± 17.43) Range: (0.0; 91.0) N = 47	16.06 (± 19.18) Range: (0.0; 80.0) N = 34	0.041
Factor influencing residents involvement in LA: BMI > 30 Yes No	6 (12.77%) 41 (87.23%) N = 47	7 (19.44%) 29 (80.56%) N = 36	0.544
Factor influencing residents involvement in LA: immudeficiency Yes No	2 (4.26%) 45 (95.74%) N = 47	3 (8.33%) 33 (91.67%) N = 36	0.648
Factor influencing residents involvement in LA:ASA > 2 Yes No	1 (2.13%) 46 (97.87%) N = 47	2 (5.56%) 34 (94.44%) N = 36	0.576
Factor influencing residents involvement in LA: Alvarado or AIR score > 2 Yes No	3 (6.38%) 44 (93.62%) N = 47	5 (13.89%) 31 (86.11%) N = 36	0.284
Factor influencing residents involvement in LA: Radiological suspicion of complicated appendicitisYesNo	17 (36.17%)30 (63.83%)N = 47	14 (38.89%)22 (61.11%)N = 36	0.98
Factor influencing residents involvement in LA: Septic shock Yes No	26 (55.32%) 21 (44.68%) N = 47	21 (58.33%) 15 (41.67%) N = 36	0.959
Factor influencing residents involvement in LA: Previous laparoscopic surgery Yes No	4 (8.51%) 43 (91.49%) N = 47	1 (2.78%) 35 (97.22%) N = 36	0.382
Factor influencing residents involvement in LA: Previous open surgery Yes No	12 (25.53%) 35 (74.47%) N = 47	13 (36.11%) 23 (63.89%) N = 36	0.424
Factor influencing residents involvement in LA: None of the previous factor is important Yes No	13 (27.66%) 34 (72.34%) N = 47	9 (25.0%) 27 (75.0%) N = 36	0.983
Factor influencing residents involvement in LA:Gangrene or Perforation Yes No	12 (25.53%) 35 (74.47%) N = 47	3 (8.33%) 33 (91.67%) N = 36	0.05
Factor influencing residents involvement in LA: Single abscess Yes No	0 (0.0%) 47 (100.0%) N = 47	1 (2.78%) 35 (97.22%) N = 36	0.434
Factor influencing residents involvement in LA:Localized Peritonitis Yes No	1 (2.13%) 46 (97.87%) N = 47	1 (2.78%) 35 (97.22%) N = 36	> 0.999
Factor influencing residents involvement in LA:Diffuse peritonitis Yes No	18 (38.3%) 29 (61.7%) N = 47	15 (41.67%) 21 (58.33%) N = 36	0.933
Factor influencing residents involvement in LA: Adhesions Yes No	14 (29.79%) 33 (70.21%) N = 47	10 (27.78%) 26 (72.22%) N = 36	> 0.999
None of the previous mentioned factor Yes No	20 (42.55%) 27 (57.45%) N = 47	14 (38.89%) 22 (61.11%) N = 36	0.911
Enviroment Factor influencing residents involvement in LA: Night time Yes No	2 (4.26%) 45 (95.74%) N = 47	2 (5.56%) 34 (94.44%) N = 36	> 0.999
Enviroment Factor influencing residents involvement in LA:Weekend Yes No	0 (0.0%) 47 (100.0%) N = 47	1 (2.78%) 35 (97.22%) N = 36	0.434
Enviroment Factor influencing residents involvement in LA: Busy operating list Yes No	8 (17.02%) 39 (82.98%) N = 47	9 (25.0%) 27 (75.0%) N = 36	0.419
Enviroment Factor influencing residents involvement in LA: Nursing staff 0t trained for laparoscopy Yes No	2 (4.26%) 45 (95.74%) N = 47	3 (8.33%) 33 (91.67%) N = 36	0.648
Enviroment Factor influencing residents involvement in LA: Unavailable basic laparoscopic instrumentary Yes No	8 (17.02%) 39 (82.98%) N = 47	8 (22.22%) 28 (77.78%) N = 36	0.585
Enviroment Factor influencing residents involvement in LA: Teaching is 0t a mission of my hospital Yes No	1 (2.13%) 46 (97.87%) N = 47	0 (0.0%) 36 (100.0%) N = 36	> 0.999
None of the above mentioned factors are important Yes No	32 (68.09%) 15 (31.91%) N = 47	21 (58.33%) 15 (41.67%) N = 36	0.493
NTS Factor influencing residents involvement in LA: Trustworthiness (reliable, honest, truthful) Yes No	28 (59.57%) 19 (40.43%) N = 47	25 (69.44%) 11 (30.56%) N = 36	0.486
NTS Factor influencing residents involvement in LA: Professional behaviour Yes No	28 (59.57%) 19 (40.43%) N = 47	28 (77.78%) 8 (22.22%) N = 36	0.129
NTS Factor influencing residents involvement in LA: Being on time Yes No	11 (23.4%) 36 (76.6%) N = 47	17 (47.22%) 19 (52.78%) N = 36	0.041
NTS Factor influencing residents involvement in LA: Recognizes is own limits Yes No	38 (80.85%) 9 (19.15%) N = 47	31 (86.11%) 5 (13.89%) N = 36	0.57
NTS Factor influencing residents involvement in LA: Ability to communicate with other healt care providers Yes No	22 (46.81%) 25 (53.19%) N = 47	21 (58.33%) 15 (41.67%) N = 36	0.412
NTS Factor influencing residents involvement in LA: Accepts negative feedbacks Yes No	30 (63.83%) 17 (36.17%) N = 47	29 (80.56%) 7 (19.44%) N = 36	0.155
NTS Factor influencing residents involvement in LA: Able to listen and follow advices Yes No	46 (97.87%) 1 (2.13%) N = 47	34 (94.44%) 2 (5.56%) N = 36	0.576
NTS Factor influencing residents involvement in LA: Kowledge of patient's history Yes No	28 (59.57%) 19 (40.43%) N = 47	26 (72.22%) 10 (27.78%) N = 36	0.334
NTS Factor influencing residents involvement in LA: Knowledge of surgical anatomy Yes No	43 (91.49%) 4 (8.51%) N = 47	34 (94.44%) 2 (5.56%) N = 36	0.693
NTS Factor influencing residents involvement in LA: Knowledge of surgical technique Yes No	40 (85.11%) 7 (14.89%) N = 47	34 (94.44%) 2 (5.56%) N = 36	0.287
NTS Factor influencing residents involvement in LA: Is updated on the most recent literature Yes No	12 (25.53%) 35 (74.47%) N = 47	13 (36.11%) 23 (63.89%) N = 36	0.424
None of the previous factor is important Yes No	0 (0.0%) 47 (100.0%) N = 47	1 (2.78%) 35 (97.22%) N = 36	0.434
Technical skills 1 2 3	15 (31.91%) 24 (51.06%) 8 (17.02%) N = 47	12 (33.33%) 17 (47.22%) 7 (19.44%) N = 36	0.933
0n Technical skills 1 2 3	26 (56.52%) 14 (30.43%) 6 (13.04%) N = 46	18 (51.43%) 14 (40.0%) 3 (8.57%) N = 35	0.613
Environmental factors 1 2 3	4 (8.7%) 9 (19.57%) 33 (71.74%) N = 46	6 (16.67%) 5 (13.89%) 25 (69.44%) N = 36	0.485
Have you ever heard about the Zwisch Scale? Yes No	4 (8.51%) 43 (91.49%) N = 47	8 (22.22%) 28 (77.78%) N = 36	0.115
Do you feel that a perioperative feedback system would increase the awareness on residents operative auto0my? (e.g., after each surgical procedure the resident and the attending fill a dedicated f 3 4 5	5 (10.64%) 13 (27.66%) 29 (61.7%) N = 47	2 (5.56%) 18 (50.0%) 16 (44.44%) N = 36	0.11

## Discussion

This prospective multicenter study examined surgical trainee involvement in laparoscopic appendectomy (LA) across the University of Milan's general surgery residency program. Analysing 653 procedures across 24 hospitals, we found that residents approached LA as primary operators in 35.9% of cases. This finding provided a foundation for exploring the dynamics of resident participation in LA as an index procedure.

We first sought to understand if there was any difference between LA's approach by residents and surgeons. The analysis disclosed three areas of discussion regarding: first, the importance of the training environment; second, clinical outcomes and training opportunities; third, adherence to guidelines and the impact of complicated diseases.

### Training environments

Academic hospitals and emergency surgery units may have played an impactful role in facilitating trainee involvement. Surgical trainees more frequently performed LA in centers with academic affiliations or an emergency surgery focus. These settings provided broader exposure to diverse cases, supporting Shetty et al.'s [16] emphasis on realistic training tools. While centers committed to surgical education showed a greater inclination toward trainee participation providing graded exposure and autonomy [2], reported barriers—including limited simulator access and curriculum gaps [4, 16] —persist also in the Italian system. These findings mirror challenges reported globally, and reinforce Wczysla et al.'s [17] call for structured, competency-based training to better prepare residents for independent practice. There has already been a call for action addressing the main critical areas of improvement, but still, no roadmap is available [7].

### Clinical outcomes and training opportunities

Procedures performed by trainees were associated with longer operative times, consistent with the learning curve documented in previous studies [3, 18]. The time required reflects the educational value of these surgeries, allowing trainees to develop technical proficiency. Despite longer durations, postoperative complication rates were lower for all complication above Clavien Dindo 1, on the other hand, no significant difference was deployed for major complications requiring invasive management. The significant difference in minor complication may be explained by lower rates of high-grade appendicitis with lower contamination performed by residents. Although the safety of trainee-performed LA under supervision was already reported by Ussia et al. [1] and Yamamoto et al. [19], the absence of a standardized approach providing graded autonomy could be could have limited residents’ involvement and crucial opportunities for surgical education in our cohort.

### The adherence to guidelines and the impact of complicated disease

Laparoscopic appendectomies approached by trainees demonstrated superior compliance with evidence-based guidelines, including peritoneal irrigation, drainage, and postoperative antibiotic protocols. These differences in peri and intra-operative attitudes may be influenced by the higher rates of complicated disease operated on by surgeons. As previously disclosed in our research [20], the higher exposure of surgeons to more complex cases may have certainly limited their adherence to guidelines, due to the psychological impact of a more complex and stressful procedure.

This enhanced adherence among residents may likely benefit from recent education, and highlights the value of integrating current guidelines into training [20, 21]. Research shows better guideline compliance in academic and emergency surgery units [20], where residents more frequently perform LA. Notably, these cases showed shorter hospital stays—contrasting with previous studies reporting longer stays with resident involvement [2]. This improvement may reflect optimal postoperative antibiotic management and the reduced use of drainages, which our earlier research linked to better outcomes and reduced healthcare costs, especially in non-complicated diseases [20]. As evidence-based guidelines recommend, there is no demonstrated advantage in the usage of peritoneal irrigation and drainages, also in complicated appendectomies. Despite this, we know that compliance with guidelines is still low [19, 20].

Finally, with the multivariable analysis, we aimed to understand which preoperative factors, linked to the clinical status and pathway of the patient could impact residents' involvement in LA. Then we explored the results of the survey to understand our research findings in relationship to residents' and surgeons' reported perceptions of the decision-making process regarding the choice of the operator. These analyses showed an interesting discrepancy. The American Society of Anaesthesiologists (ASA) score emerged as the primary clinical factor independently limiting trainee participation. This data reflects a safety-conscious approach by surgeons in charge of the decision-making of the operator, aligned with research supporting experience-matched case complexity [5]. On the other hand, the survey revealed that surgeons did not declare to prioritize clinical factors, giving instead priority to soft skills, like reliability and punctuality, over clinical ones during the choice of the operator, whilst residents agreed that case complexity could be a participation barrier. This disparity highlights the need for standardized processes and structured closed-loop feedback systems to bridge perception gaps between residents and surgeons [22] and allows trainees to be gradually exposed to more complex procedures.

### Study significance and educational implications

Early involvement in laparoscopic appendectomy enables residents to approach minimally invasive techniques, with studies [19, 23] showing comparable outcomes regardless of open surgery experience. Task-specific simulations and feedback systems enhance skill acquisition and confidence [16, 22], a point supported by both surgeons and residents in our survey. Moreover, this approach could help the surgeons, who are always in charge of the choice of the operator in Italy, to limit the effect of environmental and clinical factors on the decision-making process. Surgeons should be able and free to apply graded autonomy models, where supervision adapts only to case complexity and trainee competency [2].

### Limitations and future directions

The observational design limits causal inferences, and its focus on a single residency network may restrict generalizability to the whole country. Moreover, concerning the results on the impact of academic centers on residents’ involvement, the generalizability could be partially affected by the predominant contribution to data collection by a few large referral centers with a structured and renewed approach to surgical education. This could be read as a limitation but also as a relevant aspect to be considered as an example that systematic residents’ involvement in LA is feasible if logistical, environmental, and cultural constraints are correctly addressed.

Another limitation of our study is the potential for clustering bias due to its multicenter design. While we attempted to mitigate this by standardizing data collection, we recognize that unmeasured variability between hospitals—such as teaching culture, operative volume, or supervisory norms—could impact resident involvement. Future studies should consider hierarchical modelling from the outset and possibly stratify results by hospital or cluster-specific characteristics to better capture and understand these contextual influences.

Furthermore, part of the data collection period fell into the first outbreak of the COVID-19 pandemic, and most of the hospitals involved were located in the core area of the outbreak. Data collection was certainly hampered by the pandemic, explaining why the number of patients enrolled does not fully reflect the true number of patients suffering from acute appendicitis who could have been hospitalized and treated during the study period. Despite this, a huge effort was made to maintain data collection.

## Conclusion

Our study demonstrates that organizational, environmental, clinical, and subjective factors can play a crucial role in the educational experience with LA of trainees and surgeons, with much room for improvement especially in general non-academic hospitals. Structured surgical educational pathways for trainees could improve adherence to guidelines, reduce length of stay, and may deliver comparable clinical outcomes to experienced surgeons. To maximize the benefits of training, future efforts should focus on closing curricular gaps, improving access to simulators, and standardizing competency-based models. By prioritizing these strategies, we can better prepare surgical residents for the challenges of independent practice while maintaining high standards of patient care.

## Supplementary Information

Below is the link to the electronic supplementary material.

Supplementary file1 (DOCX 25 KB)

Supplementary file2 (DOCX 165 KB)

## Data Availability

The datasets generated during and/or analyzed during the current study are available from the corresponding author upon reasonable request.

## References

[1] Ussia A, Vaccari S, Gallo G et al (2021) Laparoscopic appendectomy as an index procedure for surgical trainees: clinical outcomes and learning curve. Updates Surg 73:187–195 33398773 10.1007/s13304-020-00950-z

[2] Young N, Ahl Hulme R, Forssten MP et al (2023) Graded operative autonomy in emergency appendectomy mirrors case-complexity: surgical training insights from the snapappy prospective observational study. Eur J Trauma Emerg Surg 49:33–44 36646862 10.1007/s00068-022-02142-3

[3] Anyomih TT, Jennings T, Mehta A et al (2023) Systematic review and meta-analysis comparing perioperative outcomes of emergency appendectomy performed by trainee vs trained surgeon. Am J Surg 225:168–179 35927089 10.1016/j.amjsurg.2022.07.006

[4] Lee W, Park SJ, Park M-S, Lee KY (2018) Impact of resident-performed laparoscopic appendectomy on patient outcomes and safety. J Laparoendosc Adv Surg Tech A 28:41–46 29016218 10.1089/lap.2017.0357

[5] Skjold-Ødegaard B, Ersdal HL, Assmus J, Søreide K (2024) Internal and external factors affecting the performance score of surgical trainees doing laparoscopic appendectomy: a prospective, observational cohort study in a structured training programme. Surg Endosc 38:4939–4946 38977503 10.1007/s00464-024-11007-2 PMC11362477

[6] Cioffi SPB, Benuzzi L, Herbolzheimer M et al (2024) Identifying and addressing mentorship gaps in European trauma and emergency surgical training. results from the Young European Society of Trauma and Emergency Surgery (yESTES) mentorship survey. Eur J Trauma Emerg Surg 50:2539–2549 39120653 10.1007/s00068-024-02610-y PMC11599355

[7] Montorsi M, De Manzini N (2019) The General Surgery Residency Program in Italy: a changing scenario. Updates Surg 71:195–196 31280461 10.1007/s13304-019-00672-x

[8] SIC - Societ Italiana di Chirurgia. https://www.sicplus.it/03_formazione/formazione_0.html. Accessed 10 Dec 2024

[9] Checklists. In: STROBE. https://www.strobe-statement.org/checklists/. Accessed 10 Dec 2024

[10] Cioffi SPB, Altomare M, Spota A et al (2019) REsiDENT 1 (Re-assessment of Appendicitis Evaluation during laparoscopic appendectomy: do we end a non-standardized treatment approach and habit?): peritoneal irrigation during laparoscopic appendectomy-does the grade of contamination matter? a prospective multicenter resident-based evaluation of a new classification system. World J Emerg Surg 14:25 31164914 10.1186/s13017-019-0243-4 PMC6543631

[11] Cioffi SPB, Granieri S, Scaravilli L et al (2023) Surgeons’ attitudes during laparoscopic appendectomy: do subjective intraoperative assessments affect the choice of peritoneal irrigation? a spin-off analysis from the REsiDENT-1 multicentre prospective observational trial. Surg Endosc 37:729–740 36307601 10.1007/s00464-022-09674-0

[12] Ferguson LW (1941) A study of the likert technique of attitude scale construction. J Soc Psychol 13:51–57

[13] Eysenbach G (2004) Improving the quality of Web surveys: the checklist for reporting results of internet E-Surveys (CHERRIES). J Med Internet Res 6:e34 15471760 10.2196/jmir.6.3.e34 PMC1550605

[14] Alseidi AA, Haukoos JS, de Virgilio C (2024) Practical guide to survey research in surgical education. JAMA Surg 159:341–342 38170507 10.1001/jamasurg.2023.6678

[15] Harris PA, Taylor R, Thielke R et al (2009) Research electronic data capture (REDCap)–a metadata-driven methodology and workflow process for providing translational research informatics support. J Biomed Inform 42:377–381 18929686 10.1016/j.jbi.2008.08.010 PMC2700030

[16] Shetty S, Zevin B, Grantcharov TP et al (2014) Perceptions, training experiences, and preferences of surgical residents toward laparoscopic simulation training: a resident survey. J Surg Educ 71:727–733 24794063 10.1016/j.jsurg.2014.01.006

[17] Wczysla K, Sparn M, Schmied B et al (2024) There is a need for a paradigm shift in laparoscopic surgical training: results of a nationwide survey among teaching hospitals in Switzerland. BMC Med Educ 24:205 38413927 10.1186/s12909-024-05209-4 PMC10900659

[18] Jolley J, Lomelin D, Simorov A et al (2016) Resident involvement in laparoscopic procedures does not worsen clinical outcomes but may increase operative times and length of hospital stay. Surg Endosc 30:3783–3791 26585194 10.1007/s00464-015-4674-z

[19] Yamamoto R, Mokuno Y, Matsubara H et al (2019) Feasibility and safety of laparoscopic appendectomy performed by residents with no experience in open appendectomy. JMA J 2:54–59 33681513 10.31662/jmaj.2018-0037 PMC7930708

[20] Cioffi SPB, Altomare M, Podda M et al (2023) The burden of the knowledge-to-action gap in acute appendicitis. Surg Endosc 37:9617–9632 37884735 10.1007/s00464-023-10449-4 PMC10709474

[21] Bass GA, Mohseni S, Ryan ÉJ et al (2023) Clinical practice selectively follows acute appendicitis guidelines. Eur J Trauma Emerg Surg 49:45–56 36719428 10.1007/s00068-022-02208-2 PMC9888346

[22] Lovasik BP, Fay KT, Patel A et al (2022) Development of a laparoscopic surgical skills simulation curriculum: enhancing resident training through directed coaching and closed-loop feedback. Surgery 171:897–903 34521515 10.1016/j.surg.2021.08.020

[23] Hiramatsu K, Toda S, Tate T et al (2018) Can laparoscopic appendectomy be safely performed by surgical residents without prior experience of open appendectomy? Asian J Surg 41:270–273 28139339 10.1016/j.asjsur.2016.12.003

