# Supplementary material for: Practice patterns and factors influencing surgical trainees’ involvement in laparoscopic appendectomy in Northern Italy's largest educational network
Source: Updates Surg. 2025 May 16;77(5):1421–37. doi: 10.1007/s13304-025-02224-y (PMC12420839; doi:10.1007/s13304-025-02224-y)
Supplement: Supplementary file 2 — Supplementary file2 (DOCX 165 KB) [file 13304_2025_2224_MOESM2_ESM.docx]

What is your role? Attending surgeon

Professor of surgery Chief of a surgical unit General surgery resident


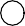

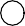

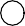

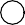

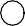

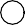

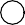

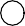

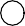


What residency year are you in? First Second Third Fourth Fifth

How long have you been in practice? Less than 5 years Between 5 and 10 years

Between 10 and 15 years


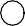

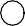

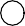

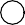

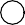


Between 15 and 20 years More than 20 years

How many laparoscopic appendectomies have you Less than 5


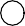

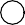

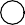

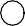

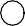


performed as first surgeon? 6 to 10

11 to 15

16 to 20

More than 20

In which kind of hospital do you work? Community hospital Large referral centre

Large referral university centre


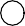

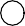

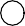

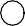

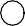


Does you hospital have a dedicate emergency surgery Yes service? No

Do you believe that the year of residency is a determining factor to let a resident operate on a

Laparoscopic Appendectomy? Non important Indifferent Very important

*(Place a mark on the scale above)*

How important do you think is the number of surgeries previously performed by a resident to be considered

ready to perform a laparoscopic appendectomy? Non important Indifferent Very important

*(Place a mark on the scale above)*

Do you think that a Laparoscopic Appendectomy should be performed by a resident only if s/he has performed a certain number of laparoscopic procedures other than laparoscopic appendectomy?


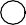
 Yes
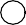
 No

How many laparoscopic procedures should have been Less than 5 performed by a resident to safely involve her/him into 5 to 10


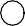

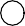

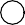

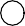

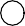


a Laparoscopic Appendectomy? 11 to 15

16 to 20

More than 20

How many laparoscopic appendectomies should a surgeon Less than 20 have performed to be ready to assist a resident during 20

a laparoscopic appendectomy? 21-30


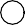

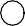

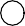

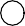

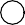

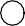

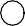


31-40

41-50

more than 50

The number doesn't matter

Do you believe that only >40 years old surgeons should Neither agree nor

assist a resident during a laparoscopic appendectomy? Strongly disagree disagree Strongly agree

*(Place a mark on the scale above)*

Which of the following patient's related factors are
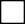
 BMI > 30 important to decide that the attending should perform
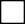
 Immunodeficiency the laparoscopic appendectomy and not the resident?
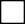
 ASA > 2


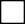
 Alvarado or AIR score > 8


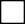
 Radiological suspicion of complicated appendicitis (gangrenous, perforated, intrabdominal abscess, peritonitis)


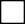
 Presence of septic shock


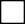
 Previous laparoscopic abdominal surgical procedures
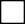
 Previous open abdominal surgical procedures


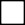
 None of the above-mentioned factors are important (more than one answer is possible)

Which of the following intraoperative findings are
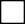
 Gangrenous or perforated appendicitis important to decide that the attending should perform
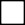
 Presence of a single abscess

the laparoscopic appendectomy and not the resident?
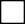
 Presence of localized peritonitis


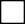
 Presence of a diffuse peritonitis
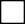
 Presence of adhesions


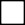
 None of the above-mentioned factors are important (more than one answer is possible)

Which of the following environment related factors are
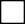
 Night time important to decide that the attending should perform
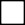
 Weekend

a laparoscopic appendectomy and not the resident?
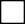
 Busy operating list


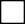
 Nursing staff not trained for laparoscopic surgery
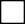
 Unavailable basic laparoscopic energy devices

(monopolar hook or bipolar forceps)


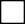
 Mine is a non-academic hospital so residents' training is not part of our mission


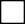
 None of the above-mentioned factors are important (more than one answer is possible)

Which of the following non-technical skills of a
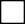
 Trustworthiness (reliable, honest, truthful) general surgery resident are important to decide that
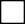
 Professional behaviour

s/he should performs the laparoscopic appendectomy and
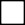
 Being on time

not the attending?
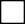
 Recognizes own limits


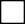
 Ability to communicate with other health care providers


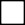
 Accepts negative feedbacks


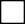
 Able to listen to and follow surgeon's intraoperative suggestions


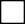
 Knowledge of patient's history


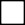
 Knowledge of surgical anatomy


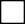
 Knowledge of surgical technique


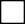
 Knowledge of the most recent evidence on the topic


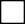
 None of the above-mentioned factors are important
(more than one answer is possible)

**How important are the following factors to decide that a general surgery resident should perform a laparoscopic appendectomy and not the attending?**

the most important medium importance the least important


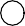

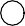

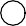
Technical skills


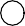

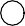

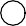

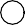

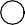

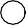
Non Technical skills Environmental factors

Do you feel that a perioperative feedback system would increase the awareness on residents operative autonomy?

(e.g., after each surgical procedure the resident and the attending fill a dedicated feedback form to assess

the operative autonomy level and personal attitude Neither agree nor

during the procedure) Strongly disagree disagree Strongly agree

*(Place a mark on the scale above)*

At the end of a laparoscopic appendectomy, do you Never

discuss with your attending about the procedure? Almost never Sometimes Almost always Always


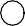

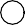

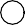

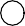

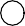


At the end of a laparoscopic appendectomy, do you Never

discuss with the resident about the procedure? Almost never Sometimes Almost always Always


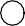

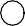

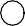

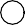

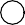


Do you usually understand the reasons why an attending Never surgeon decides to step in or directly perform a Almost never

laparoscopic appendectomy? Sometimes Almost always Always


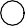

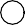

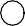

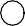

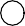


Following the last question, do you usually agree with Never

the choice? Almost never


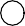

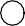

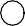

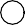


Sometimes Almost always Always

Supplementary material S2. Web-based survey exploring residents’ and surgeons’ perceptions of the decision-making process leading to the choice of the operator of a laparoscopic appendectomy.
